# A Corneal Perforation Related to Beauveria Bassiana and Post-Penetrating Keratoplasty Management Discussion

**DOI:** 10.7759/cureus.15724

**Published:** 2021-06-17

**Authors:** Spyros Atzamoglou, Maria Siopi, Joseph Meletiadis, Ioannis Markopoulos, Loukas Kontomichos, George Batsos, Efstratios Paroikakis, Vasileios Peponis

**Affiliations:** 1 Ophthalmology, Ophthalmiatreio Eye Hospital, Athens, GRC; 2 Microbiology, Attikon University Hospital, Athens, GRC

**Keywords:** fungal, keratitis, penetrating keratoplasty, beauveria bassiana, filamentous

## Abstract

Fungal keratitis is an infection that is insidious and frequently misdiagnosed. Those with chronic eye surface conditions, contact lenses, systemic immunosuppression, and diabetes have been the most frequently affected with fungal keratitis. An 84-year-old male patient with a history of bilateral penetrating keratoplasty (PK) for keratoconus presented with pain and decreased visual acuity on his left eye. A corneal perforation was found, which was treated immediately with a full-thickness corneal transplant. The specimen was sent for bacterial and fungal cultures. Topical corticosteroids were prescribed postoperatively. *Beauveria bassiana* was isolated from the corneal scrapings. The postoperative treatment was modified by reducing the dose of corticosteroid and adding topical natamycin together with systemic posaconazole. No recurrence occurred in the transplant four months postoperatively under topical dexamethasone 0.1% b.i.d.

This is the first case of keratitis and perforation in a previously transplanted cornea. Due to the rarity of the infection, there are no clear guidelines for postoperative prophylaxis in *B. bassiana* infection. Either the continuation of corticosteroids or the switch to another immunosuppressive therapy and selecting the appropriate antifungal regimen posed a significant therapeutic dilemma.

## Introduction

Fungal keratitis is an insidious and commonly misdiagnosed infection. The subjects more commonly affected with fungal keratitis include those with chronic ocular surface disease, contact lens wearers, systemic immunosuppression, and diabetes. Filamentous keratitis is often associated with minor ocular trauma, involving plant matter or agricultural tools [1]. *Beauveria bassiana* is an opportunistic filamentous fungus ubiquitous in soil and is widely used as biopesticide due to its entomopathogenic properties [2]. It is an infrequent cause of infectious keratitis. We present a case of corneal perforation from *B. bassiana*, treated with emergency penetrating keratoplasty and discuss the options in post-operative management.

## Case presentation

An 84-year-old Caucasian man, living in the countryside, presented with ocular pain and redness on his left eye. A central corneal perforation of unknown etiology was noted. The patient’s past medical history included diabetes mellitus type II and systemic hypertension, treated with metformin and enalapril, respectively. The primary penetrating keratoplasty for advanced keratoconus had been performed on both eyes 10 years earlier.

According to the referring ophthalmologist, six months before presentation, the patient had been followed elsewhere. He had developed a slow progressive thinning of the left graft with no corneal or anterior chamber inflammation signs. A therapeutic contact lens had been placed and topical chloramphenicol 0.5% (w/v) twice a day had been given as chemoprophylaxis. The patient presented to our institute two weeks after the onset of ocular pain and irritation. The visual acuity was counting fingers and slit-lamp biomicroscopy revealed an impending central corneal perforation with descemetocele formation. A localized anterior stromal inflammation was present surrounding the perforation site. The patient mentioned daily agricultural activities without any ocular trauma. Emergency penetrating keratoplasty (PK) was performed with a recipient trephination of 7.75 mm and a donor trephination of 8.00 mm. The patient’s corneal specimen was sent for bacterial and fungal cultures.

Microbiology

**Figure 1:**
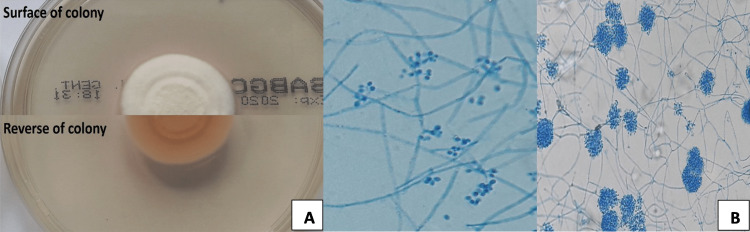
Macroscopic (A) and microscopic (B) morphologies of Beauveria​​​​​​​ bassiana isolated from the scraped cornea after eight days of incubation at 30°C. (Lactophenol cotton blue staining of slide culture, original magnification 400x.)

A direct smear examination of corneal scrapings with calcofluor white fluorescent staining did not show the presence of fungal elements. Corneal scrapings were inoculated onto blood agar, MacConkey agar, chocolate agar, and thioglycolate broth (Oxoid) incubated at 37°C, as well as Sabouraud glucose agar with gentamicin and chloramphenicol (Oxoid) incubated at 30°C. After four days of incubation, no recovery of bacteria and fungi was observed on plates incubated at 37°C, while a mold was isolated from medium incubated at 30°C. The fungus was identified as *B. bassiana* based on its colonial and microscopic features, while it could not be considered as a culture contaminant since the clinical specimen was collected under aseptic conditions and Beauveria spp. is not a component of the human skin flora. The colonies grown on Sabouraud glucose agar were slightly raised, cottony with a powdery surface lightly downy with circular rings, at first white, but later becoming pale yellow (Figure 1A). Lactophenol cotton blue staining revealed that small (<4 μm diameter), single-celled, round to oval, smooth-walled conidia were produced from small pegs formed in a zig-zag arrangement at the tips of the conidiogenous cells, which were slightly swollen at the base and were aggregated in moderately dense clusters alongside the narrow hyphae (Figure 1B). The isolate was subjected to confirmatory molecular identification by sequencing the ITS1-5.8S-ITS2 region as previously described using ITS1 (5-TCCGTAGGTGAACCTGCGG-3) and ITS4 (5-TCCTCCGCTTATTGATATGC-3) primers. A high sequence alignment (>99%) was found in GenBank Blast analysis with *B. bassiana* (GenBank accession no. MT912079).

Given the inability of the recovered strain to grow at 37°C, antifungal susceptibility testing was performed following the Clinical and Laboratory Standards Institute (CLSI) broth microdilution reference methodology (M38) after incubation at 30°C for 72h, as previously reported [3-6]. The minimum inhibitory concentration (MIC) values were 2 mg/L for amphotericin B, >64 mg/L for fluconazole, 4 mg/L for voriconazole and isavuconazole, 0.25 mg/L for itraconazole and posaconazole, and >8 mg/L for flucytosine. Echinocandin's minimum effective concentration (MEC) were 0.25 mg/L for anidulafungin and 0.125 mg/L for micafungin. The isolate was stored (normal saline with 10% glycerol, -70°C) in the culture collection of the Clinical Microbiology Laboratory’s Mycology Unit of “Attikon” University Hospital (Athens, Greece) as AUH1800.

**Figure 2:**
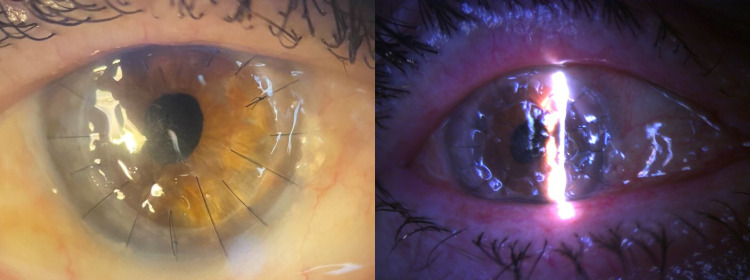
Four months post-operatively, the graft is clear with no signs of peripheral infiltration and a mild central subepithelial haze (left). In comparison, seven days post-operatively mild epithelial defect is detected (right).

The post-penetrating keratoplasty medication was initially topical dexamethasone 0.1% every one hour and chloramphenicol 0.5% every four hours. The microbiology result was issued seven days post-operatively, and the treatment was switched to topical dexamethasone 0.1% twice a day with the addition of topical natamycin 5% twice a day along with the oral solution of posaconazole 100 mg twice a day as antifungal therapy based on the in vitro susceptibility profile of the recovered isolate. A low dose of posaconazole was preferred as our patient was immunocompetent and there were no signs of peripheral corneal infiltration. The patient presented with elevated blood glucose level and liver enzymes one week after the initiation of posaconazole (morning glucose level 175 mg/dl, SGOT 45 U/L, and SGPT 30 U/L from baseline levels of 130 mg/dl, 31 U/L, and 17 U/L respectively). The oral treatment was switched to voriconazole 200 mg every 12 hours along with topical natamycin and both were discontinued after one month. Four months post-operatively, currently under topical dexamethasone 0.1% twice a day, the corneal graft is clear, with no signs of margin infiltration (Figure 2).

## Discussion

*B. bassiana* is a rare cause of human infection. In particular, only four cases of opportunistic infections in immunocompromised patients have been documented, while 15 case reports of infectious keratitis associated with ocular trauma or contact lenses use have been published since 1984 [2-5,7]. Only three cases have been reported in European countries and all three in Mediterranean countries (Italy, Spain, Portugal). It was previously reported that *B. bassiana* could not grow at ≥35°C, which agrees with our findings [2-4,8-10]. Since the ability to grow at 35-37°C is considered a requirement for human pathogenicity, the temperature sensitivity might explain why the infections in humans are limited to the body surface tissues, such as the cornea.

Although our patient denied any ocular trauma or contact with biopesticides, his daily agrarian activities increase the risk of such exposures. Diabetes mellitus may suggest a compromised immune system making opportunistic infections such as *B. bassiana* more likely.

Previous reports suggesting slow-progressing corneal thinning with superficial corneal involvement and late penetration in *B. bassiana* keratitis are consistent with our findings [9,11]. To our knowledge, this is the first case report of corneal perforation on a full-thickness corneal transplant due to *B. bassiana*.

No treatment guidelines specific to *B. bassiana* exist; thus, post-penetrating keratoplasty management was based on guidelines for filamentous fungi and the antifungal susceptibility testing results. A lower dosage of topical dexamethasone 0.1% was preferred to cyclosporin or other immunomodulatory agents due to high postoperative inflammation. The oral suspension of posaconazole was added based on susceptibility testing results. Because our patient developed side effects that resolved with discontinuation of the drug, oral voriconazole was given based on susceptibility test results and published literature, as aforementioned [12,13]. Systemic antifungal therapy combined with topical natamycin was preferred to topical treatment alone due to the extended use of dexamethasone before the results of cultures were available. Among systemic antimold drugs, voriconazole is detectable in the aqueous and vitreous humor of animal and human eyes at 40-100% of serum levels, whereas lower posaconazole concentration has been described in the literature [14].

## Conclusions

The penetrating keratoplasty in our case was the treatment for both corneal perforation and *B. bassiana* infection. The progressive thinning of the primary graft in our case, although demonstrating mild inflammation, was of infectious etiology and an early corneal culture could have been imperative for the treatment. The treatment efficacy of oral voriconazole with topical natamycin on a full-thickness corneal graft was not assessed in this case, but the administration of lower than therapeutic dosage proved adequate and recurrence in the therapeutic graft was avoided.

## References

[1] Jack Kanski, Brad Bowling (2015). Kanski’s Clinical Ophthalmology: A Systematic Approach, 8th Edition. Chapter 6, Cornea.

[2] Figueira L, Pinheiro D, Moreira R (2012). Beauveria bassiana keratitis in bullous keratopathy: antifungal sensitivity testing and management. Eur J Ophthalmol.

[3] García Lozano T, Aznar Oroval E, Pérez Ballestero P, Lorente Alegre P (2011). Isolation of Beauveria bassiana in a bronchoalveolar lavage specimen from a patient with bladder cancer. [Article in Spanish]. Rev Iberoam Micol.

[4] Gürcan S, Tuğrul HM, Yörük Y, Ozer B, Tatman-Otkun M, Otkun M (2006). First case report of empyema caused by Beauveria bassiana. Mycoses.

[5] Lara Oya A, Medialdea Hurtado ME, Rojo Martín MD (2016). Fungal keratitis due to Beauveria bassiana in a contact lenses wearer and review of published reports. Mycopathologia.

[6] Alexander BD, Procop GW, Dufresne P (2017). M38: Reference Method for Broth Dilution Antifungal Susceptibility Testing of Filamentous Fungi. 3rd edition. https://clsi.org/media/1894/m38ed3_sample.pdf.

[7] de la Presa M, McCafferty B, Page MA (2020). Refractory keratitis from Beauveria bassiana—successful treatment with intrastromal amphotericin B and micafungin. JCRO.

[8] Mitani A, Shiraishi A, Miyamoto H (2014). Fungal keratitis caused by Beauveria bassiana: drug and temperature sensitivity profiles: a case report. BMC Res Notes.

[9] Ogawa A, Matsumoto Y, Yaguchi T, Shimmura S, Tsubota K (2016). Successful treatment of Beauveria bassiana fungal keratitis with topical voriconazole. J Infect Chemother.

[10] Sonoyama H, Araki-Sasaki K, Kazama S (2008). The characteristics of keratomycosis by Beauveria bassiana and its successful treatment with antimycotic agents. Clin Ophthalmol.

[11] Tu EY, Park AJ (2007). Recalcitrant Beauveria bassiana keratitis: confocal microscopy findings and treatment with posaconazole (Noxafil). Cornea.

[12] Tucker DL, Beresford CH, Sigler L, Rogers K (2004). Disseminated Beauveria bassiana infection in a patient with acute lymphoblastic leukemia. J Clin Microbiol.

[13] Ullmann AJ, Lipton JH, Vesole DH (2007). Posaconazole or fluconazole for prophylaxis in severe graft-versus-host disease. N Engl J Med.

[14] Felton T, Troke PF, Hope WW (2014). Tissue penetration of antifungal agents. Clin Microbiol Rev.

